# Broadening Diagnostic Horizons: Specificity of Serial Negative CRPs in Predicting Blood Culture Negativity in Suspected Neonatal Sepsis

**DOI:** 10.7759/cureus.81660

**Published:** 2025-04-03

**Authors:** Gullapudi Prakash, Ritvik Sajan, Gayathri G Reshma, Georg Gutjahr, Varsha V S, M P Narmadha, Perraju Bendapudi

**Affiliations:** 1 Department of Neonatology, Amrita Institute of Medical Sciences and Research, Ernakulam, IND; 2 Department of Pharmacy Practice, Amrita Institute of Medical Sciences and Research, Ernakulam, IND; 3 Department of Mathematics, Amrita Vishwa Vidyapeetham, Kollam, IND; 4 Department of Health Science Research, Amrita Institute of Medical Sciences and Research, Ernakulam, IND; 5 Department of Paediatrics, Amrita Institute of Medical Sciences and Research, Ernakulam, IND

**Keywords:** cost-effectiveness, c –reactive protein (crp), low and middle-income countries, neonatal care, neonatal infection, neonatal intensive care unit (nicu), neonatal sepsis

## Abstract

Background

Neonatal sepsis is a root cause, among others, of mortality and morbidity among neonates in low-middle-income countries (LMICs). Blood culture remains the gold standard diagnostic tool in diagnosing sepsis; however, it may take 48 to 72 hours to be reported negative. Hence, there is a demand for a quicker alternative to blood cultures. C-reactive protein (CRP) is a potential candidate useful in ruling out sepsis and can be a potential substitute for blood cultures. This prospective study aims to identify whether serial negative CRPs in a suspected case of sepsis can predict negative blood culture and aid in the safe, early discontinuation of antibiotics.

Methods

The study included babies for whom antibiotics were started on suspicion of sepsis. Two CRP tests, 24 hours apart, were negative. Blood cultures were evaluated for growth at 48 hours and 5 days. The cost incurred with additional antibiotics after two negative CRPs was estimated.

Results

Among 100 babies enrolled (n=100), 90 (n=90) were suspected of early-onset and 10 (n=10) were suspected of late-onset sepsis. The median gestational age was 34 weeks (interquartile range (IQR): 31.75-35). The median first and second CRP values are 0.2 (IQR: 0.1-0.31) and 1.2 mg/dl (IQR: 0.4-2.02), respectively. All blood cultures (n = 100) were negative, with a 95% confidence interval of 0.96 to 1.

Conclusion

Two negative CRPs 24 hours apart are sufficient to rule out sepsis with high confidence and predict a negative blood culture for microbial growth. Thus, antibiotics can be stopped following two negative CRPs.

## Introduction

Neonatal sepsis, in term and preterm babies, is the dysregulated response of the host toward an infection, which could potentially be life-threatening, leading to multiorgan failure [1].

A large multicentric Indian study states that the total incidence of neonatal and culture-positive sepsis was 14.3% and 6.2%, respectively [2]. Nearly two-thirds of the above neonates were diagnosed with early-onset sepsis (EOS), and two-thirds of culture-positive sepsis was due to gram-negative organisms. Another study from India showed that implementing antibiotic stewardship significantly reduced mean monthly costs by 14.4% [3]. 

Antibiotics serve as essential tools in managing sepsis and are the most frequently prescribed medications in the neonatal intensive care unit (NICU) [4,5]. However, due to a concerning rise in multidrug-resistant strains among neonates in India, clinicians’ choices of antibiotics are increasingly being questioned [2]. A retrospective study revealed that approximately 35% of neonates received inappropriate antibiotics in the NICU, underscoring the necessity for antibiotic stewardship [6].

Since neonatal sepsis is defined by culture positivity, blood culture is the “gold standard” investigation for sepsis identification. However, blood culture positivity is influenced by the volume of blood collected and pretreatment with antibiotics. In preterm and low birth weight babies, getting enough blood volume for inoculation in the culture bottles can be challenging. Many of these babies might be exposed to antibiotics, either transplacental when administered to mothers or postnatally from referral centers. All these factors can greatly reduce the yield of blood culture. Diagnosing neonatal sepsis by clinical signs and symptoms is fraught with problems, as they are very subtle and often overlap with non-infectious etiology. Hence, valuable time is lost before starting antibiotics. The sensitivity and specificity of the other modalities leave significant room for improvement: C-reactive protein (CRP) (sensitivity: 49-68%, specificity: 90%) and procalcitonin (sensitivity: 81%, specificity: 79%) [7-9]. In 70% of cases of neonatal sepsis, the colony count of bacteremia is low, resulting in negative blood cultures despite other markers suggesting sepsis [10]. A meta-analysis of 23 studies, comparing traditional blood cultures to molecular methods (polymerase chain reaction), showed a 96% sensitivity and specificity in molecular methods [11]. However, the costs involved and the inability to identify the sensitivity pattern of the bacteria are major limitations of this test.

Due to its high sensitivity and minimal blood requirement, CRP is an ideal biomarker for determining neonatal sepsis. CRP levels are significant in determining sepsis. However, not in all cases, CRP is sensitive in predicting infection. In cases of coagulase-negative staphylococcal bacteremia, particularly in very preterm infants, CRP levels may not rise significantly, further contributing to false-negative results [12]. However, serial CRP values (at 24 hours and 48 hours) have improved sensitivity from 82% to 84% [13]. Ali et al. (2024) report that an increase in CRP (>10 mg/L) was associated with positive blood cultures in 55.3% of neonates with sepsis [14].

CRP can assist in overcoming the delay faced while awaiting 48-hour blood cultures. This study seeks to examine the effectiveness of two consecutive negative CRP values in predicting blood culture negativity in neonates suspected of having sepsis.

## Materials and methods

Study design and participants

This prospective single-center study was conducted at the NICU of a tertiary care hospital in India. It took place over six months, from February 2024 to July 2024.

The inclusion criteria included all neonates who underwent a partial septic screen (CRP, complete blood count (CBC), and blood culture) and were commenced on IV antibiotics at admission or upon suspicion of sepsis. The exclusion criteria consisted of neonates with a positive first or second CRP, those with admission CRPs not tested, and babies who had been exposed to antibiotics before admission.

The Ethics Committee of Amrita School of Medicine approved the study, with ethics approval number ECASM-AIMS-2024-467, prior to its conduct. As this was a prospective observational study that did not involve any new investigation or intervention, informed consent was waived.

Sample size

The objective was to select a sufficiently large sample to estimate the specificity of CRP in detecting blood culture negativity in neonates. More specifically, we aimed for a sample that would provide a 95% confidence interval for the specificity, with an expected length of no more than 0.1. We assumed that the specificity would be at least 0.95. Consequently, the minimum required sample size was calculated to be 94. 

Definitions

Early-onset sepsis and late-onset sepsis (LOS) were defined as the onset of sepsis within 72 hours and after 72 hours of birth, respectively. Positive CRP was defined as ≥ 5 mg/L while negative CRP was defined as < 5 mg/L.

Procedures

Patients enrolled in the study based on inclusion and exclusion criteria were evaluated, and patient data were recorded prospectively.

The current practice for screening suspected episodes of sepsis involves blood samples, a CBC, CRP, and blood cultures, which are collected at the time of admission (TOA) of the neonate or the initial time of suspicion (TOS). An additional CRP sample is collected after 24 hours. One milliliter of the subject’s blood is collected and sent to the laboratory for CRP measurement. The laboratory cutoff for CRP value is 5 mg/L. Blood cultures are evaluated for growth at 48 hours and again after five days. When sending these blood samples, antibiotics are prophylactically initiated (ampicillin, piperacillin-tazobactam, or cefoperazone-sulbactam alongside amikacin) as per clinical indication and unit practice guidelines, which are based on our local bacterial spectrum and clinical suspicion. The antibiotics are discontinued if both CRPs return negative, if there is resolution of the initial symptoms or signs that warrant sepsis screening, and if the blood culture reports are negative for microbial growth following 48 hours of incubation. The costs incurred for the additional doses of antibiotics after two negative CRPs are also recorded.

Data collection

All patient records were accessed through the hospital’s neonatal and electronic medical records databases. Information from maternal medical histories, neonatal admission records detailing the reasons for admission, and laboratory results obtained during the neonatal period were also utilized. Laboratory results were retrieved from the hospital database and documented in the medical records.

Additional data were obtained from admission summaries and daily progress notes that included indications for antibiotic therapy, laboratory biomarkers, CRP concentrations, blood culture results, and maternal antibiotic exposure prior to parturition.

Outcomes

The primary aim of this study was to estimate the specificity of two negative CRPs in predicting blood culture negativity in neonates suspected of sepsis. A secondary aim was to evaluate the common factors that may cause sepsis and the effect of using two negative CRPs on the total duration and cost of antibiotic therapy.

Statistical analysis

Data were collected from the NICU between February and July 2024 for all neonates who were started on antibiotics for suspected sepsis and who had two negative CRP tests conducted 24 hours apart. The study aimed to evaluate whether two negative CRPs can reliably predict culture negativity at 48 hours and five days. Specificity with a 95% confidence interval was calculated for this prediction. Descriptive statistics were employed to summarise the baseline characteristics of the study population, including median, interquartile range (IQR), maximum and minimum gestational age at sepsis, successive CRP values, white cell counts (WCC), differential cell counts (DCC), and platelets. All statistical analyses were performed using R version 4.4.0. A p-value of p<0.05 was considered statistically significant.

## Results

In a study involving 100 neonates (n=100), blood cultures were negative in every case. Table 1 presents the baseline characteristics of early preterm and preterm infants.

**Table 1:** Baseline characteristics of early preterm and preterm infants 1 - Median (IQR), 2 - Wilcoxon rank sum test CRP: C-reactive protein, WCC: white cell count, DCC: differential cell count

Characteristic	Overall, N = 100^1^	Early Preterm, N = 34^1^	Preterm, N = 66^1^	t-test Statistic	p-value^2^
Gestational Age	34.0 (31.8, 35.0)	31.0 (28.0, 31.8)	35.0 (34.0, 36.0)	-12.849	<0.001
1st CRP (mg/l)	0.2 (0.1, 0.3)	0.2 (0.1, 0.6)	0.2 (0.1, 0.3)	1.322	0.8
2nd CRP (mg/l)	1.2 (0.4, 2.0)	1.2 (0.5, 2.2)	1.2 (0.4, 2.0)	0.777	0.7
WCC	12,500.0 (10,000.0, 16,000.0)	11,000.0 (9,000.0, 14,750.0)	13,000.0 (10,000.0, 16,000.0)	-1.363	0.14
DCC Neutrophils	47.0 (38.0, 56.0)	40.0 (33.0, 46.8)	51.5 (45.0, 58.0)	-3.398	<0.001
DCC Lymphocytes	36.0 (28.0, 44.3)	43.0 (36.3, 55.5)	32.0 (26.0, 40.0)	3.566	<0.001

Gestational age is significantly lower in the early preterm group, while CRP levels and WCC show no notable differences between groups. 

Table 2 presents the demographic distribution and test specificity for early preterm and preterm infants.

**Table 2:** Demographic distribution and test specificity for the study sample CI: confidence interval

Parameter	Overall	Early Preterm	Preterm	Test Positive	Specificity	95% CI	Chi-Square Statistic	P-Value
Sample size	100	34	66	0	100%	96%-100%	2.61	0.106
Female	42	10	32	0	100%	92%-100%	11.5	0.001
Male	58	24	34	0	100%	94%-100%	1.724	0.189

The overall sample consisted of 100 participants (n=100), with no test positives observed. Specificity remains at 100% across all categories, with confidence intervals ranging from 92% to 100%. While sex-based differences are observed, with a significant association in females (χ² = 11.5, p = 0.001), no significant difference is noted in males (χ² = 1.724, p = 0.189). The overall chi-square test does not indicate a statistically significant association (χ² = 2.61, p = 0.106).

Table 3 represents the frequencies of low and normal birth weights among male and female neonates to determine whether variation in birth weight influences blood culture positivity.

**Table 3:** Frequencies of low and normal birth weights among male and female neonates, along with test specificity, confidence intervals, chi-square statistics, and p-values for group-wise comparisons

Group	Sample Size	Low Birth Weight	Normal Birth Weight	Test Positive	Specificity	95% CI	Chi-Square Statistic	P-Value
Overall	100	54	46	0	100%	96%- 100%	0.681	0.409
Female	42	22	20	0	100%	92%- 100%	0.423	0.515
Male	58	32	26	0	100%	94%- 100%	0.164	0.686

Table 4 illustrates the risk factors associated with sepsis in the study participants. The findings indicate that respiratory distress (n=85, 85%) was identified as the primary cause of sepsis among the subjects.

**Table 4:** Risk factors associated with sepsis in study subjects PV leak: para valvular leak

Risk Factors	Number of Neonates (n=100)	Percentage of Neonates (n%)
Respiratory distress	85	85%
Hypoglycaemia	6	6%
PV leak	3	3%
Abdominal distention	2	2%
Tachycardia	1	1%
Icterus	1	1%
Anuria	1	1%
Leukocytosis	1	1%

The number of neonates with suspected EOS and suspected LOS was 90 (n=90, 90%) and 10 (n=10, 10%), respectively. The greatest number of neonates were suspected of early-onset sepsis.

Table 5 depicts the additional number of antibiotic doses and the cost of each additional dose.

**Table 5:** Cost involved with additional doses of antibiotics

Antibiotic	Total number of additional doses administered	Total percentage of additional doses administered	Cost of each dose (in Rs.)	Cost of total doses (in Rs.)
Ampicillin	236	48.76%	16	3,776
Amikacin	141	29.13%	30	4,230
Piptaz (Piperacillin + Tazobactam)	79	16.32%	37.3	2,946.7
Meropenem	12	2.47%	269.6	3,235.2
Cefoperazone Sulbactam	6	1.23%	159.5	957
Cefotaxime	10	2.06%	19	190

The cost of each additional dose of ampicillin (n=236, 48.76%), amikacin (n=141, 29.13%), piperacillin-tazobactam (n=79, 16.32%), meropenem (n=12, 2.47%), cefoperazone-sulbactam (n=6, 1.23%), and cefotaxime (n=10, 2.06%) was Rupees 16, 30, 37.3, 269.6, 159.5, and 19, respectively. On average, this would amount to 153 rupees in extra costs per baby.

## Discussion

This prospective study aimed to determine the specificity of two negative CRP tests, at the TOS and after 24 hours, in predicting blood culture negativity in neonates suspected of sepsis. We found that all 100 neonates (n=100, 100%) had negative CRP results. In this study, the terms positive and negative CRP are used. A negative CRP value is defined as < 5 mg/L, while a positive CRP is defined as ≥ 5 mg/L.

Blood culture remains the gold standard for the diagnosis of sepsis in neonates, but it is hindered by limited sensitivity and several other factors elaborated above. CRP is a non-specific systemic inflammatory marker with good sensitivity but poor specificity in identifying neonatal sepsis. However, serial CRP measurements, when taken 24 hours apart in combination with blood culture, aid in safe early discontinuation of antibiotics and reduce the duration of antibiotics and cost implications [15-17].

The relationship between positive CRP and culture-positive sepsis is well-established, yet there is a lack of data concerning the use of negative CRP to predict culture-positive sepsis [15,18,19]. We identified that two consecutive negative CRPs (at the TOS and repeated at 24 hours) could reliably predict negative blood culture.

Literature indicates that the sensitivity of CRP increases over time post-birth, which is linked to the liver's capacity to produce CRP, which peaks around 48 to 72 hours after birth [20]. Benitz et al. stated that the initial CRP at the TOA is insufficient to confirm or exclude sepsis. Serial CRP measurements taken 8 to 48 hours apart from the TOA demonstrate greater sensitivity in identifying sepsis [15]. Kawamura et al. found that CRP peaks 24 hours post-birth, regardless of infection or antibiotic use [21]. Ávila et al. measured serial CRPs at 12 and 48 hours and concluded that they possess a high negative predictive value in ruling out sepsis [22].

Nonetheless, our study evaluated CRPs at TOA or TOS and after 24 hours. Our results suggest that CRP has 100% specificity (specificity: 1, confidence interval of specificity: 0.96-1.00) in ruling out culture positivity. Therefore, one can confidently assert that CRPs (TOA and at 24 hours) can rule out sepsis in newborns without waiting for blood culture reports. The possibility of birth weight influencing the blood culture positivity was ruled out as changes in birth weight were not significant (Table 3). Despite having the same objectives, different studies define their cut-off CRP values in varying ways. Studies by Benitz et al. and Kawamura et al. considered 1 mg/dl as their cutoff CRP value [15,21]. Effat et al. used 5 mg/dl as their cut-off value [20]. Ávila et al. determined a cut-off of 6 mg/L in their study according to their institutional reference value [22]. Our study employed 5 mg/L as the cut-off CRP value. Based on various results from different studies, we can infer that the CRP threshold value should be tailored to the institutional laboratory.

There is a lack of data regarding whether the CRP test used in other studies was a regular CRP test or a highly sensitive CRP (hs-CRP) test. Generally, hs-CRP has a lower measurement range (below 1 mg/L) than regular CRP [23]. Previous literature has not specified whether the CRP was regular or hs-CRP, which can lead to ambiguity in results [1,15,21,22]. Our study utilized regular CRP, with results showing the median (IQR) of the first CRP was 0.2 (IQR: 0.10-0.31) and the second CRP was 1.2 (IQR: 0.4-2.02) in all samples analyzed.

CRP is one of the various markers or diagnostic tests useful for detecting sepsis. Commonly used markers include microbiological culture methods, nucleic acid-based methods (Verigene gram-positive/gram-negative, Film Array, Gene Xpert), hematological indices (leukocyte count, white blood cell count, absolute neutrophil count, immature to total neutrophil ratio, red cell distribution width, thrombocytopenia), and inflammatory biomarkers (CRP, procalcitonin, serum amyloid A, tumor necrosis factor-α, fibronectin, haptoglobin, pro-adrenomedullin) [24]. CRP, however, offers a significant advantage compared to other diagnostic tests because it provides results in less time than other tests (such as microbial cultures), which are essential for the early diagnosis of sepsis. Additionally, CRP is cheaper than other tests, has high specificity, and requires minimal blood [21]. According to Kaur et al., CRP has the highest sensitivity (87.5%) among micro-ESR and gastric aspirate for polymorphs in diagnosing early-onset neonatal sepsis [13].

Several studies suggest a relationship between serial CRPs and sepsis, highlighting the need to properly discontinue or de-escalate antibiotics. A study by Bomela et al. in South Africa found that repeat CRPs (24 and 48 hours after birth) possess a negative predictive value of 99%, indicating that 99 out of 100 subjects do not require further antibiotic therapy [25]. However, the microbial strains in South Africa may differ from those in India. Additional studies also underscore the importance of utilizing CRP to determine antibiotic duration [21,26,27]. In our study, combinations of ampicillin, piperacillin-tazobactam, cefoperazone-sulbactam, or amikacin were initiated prophylactically. We observed that all babies with two consecutive negative CRPs subsequently had negative cultures, suggesting that stopping antibiotics would have been the correct decision. This approach could alleviate the financial burden on patients and encourage appropriate antibiotic prescribing practices, thereby reducing concerns regarding antibiotic resistance, a global issue. Nonetheless, our study subjects are from India, where the predominant bacterial strains are gram-negative, limiting the generalisability of these findings to other countries. Nevertheless, these results may hold relevance in nations facing significant gram-negative sepsis.

However, CRP cannot be deemed a conclusive test for ruling out sepsis. Hofer et al. assert that one should not depend on CRP as the sole indicator for discontinuing antibiotics in neonatal sepsis [28]. Maternal risk factors, hematological parameters, and physicians' judgment must also be considered before ceasing antibiotics [29]. Serial-negative CRPs can effectively rule out sepsis if other infection parameters support the same conclusion.

The underlying causative pathogen may influence the rise in CRP. Lai et al. identified that blood cultures of neonates with CRP ≤ 10 mg/L were infected with coagulase-negative Staphylococci (CoNS) (55.9%) [30]. Therefore, one should be aware of such scenarios and not rely solely on CRP values. This may explain the different conclusions drawn by other studies, influenced by their underlying pathogenic organisms.

Strengths and limitations

The study results suggest that clinicians can discontinue intravenous antibiotics in cases of suspected sepsis after two negative CRP tests spaced 24 hours apart, provided the initial clinical concerns indicative of sepsis are resolved. The financial burden associated with additional antibiotic doses was also calculated, which could contribute to improving patient quality of life and preventing the emergence of antibiotic resistance.

This study's limitation was that it was single-centered and conducted in a region where gram-negative organisms are more prevalent. Consequently, the results cannot be extrapolated to areas with a higher prevalence of gram-positive strains of bacteria.

## Conclusions

Serial negative CRPs and the resolution of initial clinical concerns are useful for ruling out sepsis. After two consecutive negative CRPs (≤5 mg/L), it can be anticipated that the blood culture will return negative for microbial growth. Therefore, antibiotics can be tapered or de-escalated following two serial negative CRPs, which helps improve patient outcomes.

## References

[1] Attia Hussein Mahmoud H, Parekh R, Dhandibhotla S (2023). Insight into neonatal sepsis: an overview. Cureus.

[2] Chaurasia S, Sankar J, Agarwal R (2016). Characterisation and antimicrobial resistance of sepsis pathogens in neonates born in tertiary care centres in Delhi, India: a cohort study. Lancet Glob Health.

[3] Singh S, Menon VP, Mohamed ZU (2019). Implementation and impact of an antimicrobial stewardship program at a tertiary care center in south India. Open Forum Infect Dis.

[4] Du Pont-Thibodeau G, Joyal JS, Lacroix J (2014). Management of neonatal sepsis in term newborns. F1000Prime Rep.

[5] Clark RH, Bloom BT, Spitzer AR, Gerstmann DR (2006). Reported medication use in the neonatal intensive care unit: data from a large national data set. Pediatrics.

[6] Patel SJ, Oshodi A, Prasad P (2009). Antibiotic use in neonatal intensive care units and adherence with Centers for Disease Control and Prevention 12 step campaign to prevent antimicrobial resistance. Pediatr Infect Dis J.

[7] Delanghe JR, Speeckaert MM (2015). Translational research and biomarkers in neonatal sepsis. Clin Chim Acta.

[8] Perrone S, Lotti F, Longini M (2018). C reactive protein in healthy term newborns during the first 48 hours of life. Arch Dis Child Fetal Neonatal Ed.

[9] Vouloumanou EK, Plessa E, Karageorgopoulos DE, Mantadakis E, Falagas ME (2011). Serum procalcitonin as a diagnostic marker for neonatal sepsis: a systematic review and meta-analysis. Intensive Care Med.

[10] Kellogg JA, Ferrentino FL, Goodstein MH, Liss J, Shapiro SL, Bankert DA (1997). Frequency of low level bacteremia in infants from birth to two months of age. Pediatr Infect Dis J.

[11] Pammi M, Flores A, Leeflang M, Versalovic J (2011). Molecular assays in the diagnosis of neonatal sepsis: a systematic review and meta-analysis. Pediatrics.

[12] Brown JV, Meader N, Cleminson J, McGuire W (2019). C-reactive protein for diagnosing late-onset infection in newborn infants. Cochrane Database Syst Rev.

[13] Kaur S, Singh K (2021). Early-onset neonatal sepsis: role of C-reactive protein, micro-ESR, and gastric aspirate for polymorphs as screening markers. Int J Pediatr.

[14] Ali AA, Ahmed M, Noor SK (2024). The relationship between blood culture, C-reactive protein, and neonatal sepsis: a cross-sectional study. Cureus.

[15] Benitz WE, Han MY, Madan A, Ramachandra P (1998). Serial serum C-reactive protein levels in the diagnosis of neonatal infection. Pediatrics.

[16] Cantey JB, Baird SD (2017). Ending the culture of culture-negative sepsis in the neonatal ICU. Pediatrics.

[17] Shane AL, Sánchez PJ, Stoll BJ (2017). Neonatal sepsis. Lancet.

[18] Mathers NJ, Pohlandt F (1987). Diagnostic audit of C-reactive protein in neonatal infection. Eur J Pediatr.

[19] Wagle S, Grauaug A, Kohan R, Evans SF (1994). C-reactive protein as a diagnostic tool of sepsis in very immature babies. J Paediatr Child Health.

[20] Hisamuddin E, Hisam A, Wahid S, Raza G (2015). Validity of C-reactive protein (CRP) for diagnosis of neonatal sepsis. Pak J Med Sci.

[21] Kawamura M, Nishida H (1995). The usefulness of serial C-reactive protein measurement in managing neonatal infection. Acta Paediatr.

[22] Puello Ávila AC, Cataño Villegas AE (2021). Utility of C-reactive protein in early neonatal sepsis [Article in Spanish]. Rev Chilena Infectol.

[23] Wolska A, Remaley AT (2022). CRP and high-sensitivity CRP: “what’s in a name?”. J Appl Lab Med.

[24] Celik IH, Hanna M, Canpolat FE, Mohan Pammi (2022). Diagnosis of neonatal sepsis: the past, present and future. Pediatr Res.

[25] Bomela HN, Ballot DE, Cory BJ, Cooper PA (2000). Use of C-reactive protein to guide duration of empiric antibiotic therapy in suspected early neonatal sepsis. Pediatr Infect Dis J.

[26] Franz AR, Steinbach G, Kron M, Pohlandt F (1999). Reduction of unnecessary antibiotic therapy in newborn infants using interleukin-8 and C-reactive protein as markers of bacterial infections. Pediatrics.

[27] Pourcyrous M, Bada HS, Korones SB, Baselski V, Wong SP (1993). Significance of serial C-reactive protein responses in neonatal infection and other disorders. Pediatrics.

[28] Hofer N, Zacharias E, Müller W, Resch B (2012). An update on the use of C-reactive protein in early-onset neonatal sepsis: current insights and new tasks. Neonatology.

[29] Eichberger J, Resch E, Resch B (2022). Diagnosis of neonatal sepsis: the role of inflammatory markers. Front Pediatr.

[30] Lai MY, Tsai MH, Lee CW (2015). Characteristics of neonates with culture-proven bloodstream infection who have low levels of C-reactive protein (≦10 mg/L). BMC Infect Dis.

